# Reduction in the Incidence of Urological Cancers after the Ban on Chinese Herbal Products Containing Aristolochic Acid: An Interrupted Time-Series Analysis

**DOI:** 10.1038/s41598-019-56394-y

**Published:** 2019-12-27

**Authors:** Jing-Rong Jhuang, Chun-Ju Chiang, Shih-Yung Su, Ya-Wen Yang, Wen-Chung Lee

**Affiliations:** 1 Institute of Epidemiology and Preventive Medicine, College of Public Health, National Taiwan University, Taipei, Taiwan; 2 Taiwan Cancer Registry, Taipei, Taiwan; 3 Innovation and Policy Center for Population Health and Sustainable Environment, College of Public Health, National Taiwan University, Taipei, Taiwan

**Keywords:** Cancer epidemiology, Bladder cancer, Renal cell carcinoma, Risk factors

## Abstract

Cancer is a public health problem worldwide. Taiwan has a higher incidence rate of urological cancers than many Asian countries do. Aristolochic acid has been considered a potent carcinogen. In this study, we examined whether the cessation of the sales and preparation of aristolochic acid-containing Chinese herbal products (AA-CHPs) in Taiwan contributed to a decline in the incidence rates of bladder cancer, carcinomas of the renal pelvis and other urinary organs, and kidney cancer. We conducted an interrupted time-series analysis of long-term trends in the incidence rates of the aforementioned cancers between 1995 and 2013 in Taiwan. The incidence rates of bladder cancer and carcinomas of the renal pelvis and other urinary organs decreased considerably after 2008 and 2011, respectively. Notably, these change-of-slope time points occurred after the year 2003, when a ban on AA-CHPs was imposed in Taiwan. The ban on AA-CHPs in Taiwan was possibly associated with the reduction in the incidence of bladder cancer and carcinomas of the renal pelvis and other urinary organs.

## Introduction

Cancer is a public health problem worldwide. Among all cancers, bladder cancer and cancers of the kidney and other urinary organs were the 9th and 13th most common worldwide in both sexes, respectively, in 2012^1^. In Taiwan, the age-standardized incidence rates of urological cancers in both sexes such as bladder cancer, kidney cancer, renal pelvis cancer, and ureteral cancer were 7.96, 5.09, 2.87, and 1.90 per 100,000 population, respectively, in 2012^2^; these rates were higher than those in many other Asian countries^3,4^.

Aristolochic acid, which occurs naturally in many plants belonging to genus *Aristolochia*, is frequently used in traditional Chinese herbal products. Aristolochic acid has not only been classified as a group 1 carcinogen by the International Agency for Research on Cancer^5^ but also been recognized as a potent carcinogen for bladder cancer in men^6^. Studies in many countries have successively demonstrated a positive association between aristolochic acid and urothelial cancers^7–16^. The molecular mechanisms of tumor formation induced by aristolochic acid were also discovered^17^.

Many countries have prohibited the use of aristolochic acid-containing Chinese herbal products (AA-CHPs)^18–20^. The first regulatory control of AA-CHPs in Taiwan began in 2000. The Ministry of Health and Welfare banned most AA-CHPs and their preparation in 2003. Wang *et al*. observed that the prescription frequencies of AA-CHPs in Taiwan for patients with end-stage renal disease began to decrease in 1999 and dropped to nearly zero after 2005^13^. They also noted that the incidence rate of urothelial cancer for these patients appeared to decrease after 2000; this effect was hypothesized to be associated with the ban on AA-CHPs^13,21^. To examine the hypothesis, in this study, we conducted an interrupted time-series analysis^22,23^ of long-term trends in the incidence of urological cancers in Taiwan.

## Materials and Methods

### Data source

All information of newly diagnosed malignant neoplasms from hospitals with capacities of more than 50 beds has been recorded in the Taiwan Cancer Registry Dataset since 1979. Information about every patient in the Taiwan Cancer Registry Dataset comprises demographics (sex, date of birth, and address) as well as diagnostic data (date of diagnosis, site of tumor, histopathological information of tumors, and tumor grade). To strengthen the validity, completeness, and timeliness of the Taiwan Cancer Registry Dataset, multiple verification processes were conducted, containing logical and consistency assessments, duplicate checks, and trace-back of death certificate only cases^24,25^.

### Case definition

We extracted data of patients between 1995 and 2013. Before 2001, all cases with definitive diagnosis were originally encoded according to the Field Trial Edition of the International Classification of Disease for Oncology (ICD-O-FT). Since 2002, the newest version, ICD-O-3^26^, has been used as the standard code, and each type of cancer was also coded in terms of ICD-O-3. We also included for analysis patients with invasive cancers aged at least 40 years with the topographical codes from C64 to C68. According to the classification used in the annual reports of Taiwan Cancer Registry^27^, these patients were divided into three categories: bladder cancer (C67), carcinomas of the renal pelvis and other urinary organs (C65, C66, and C68), and kidney cancer (C64).

### Statistical analysis

We assumed that the effect (if any) of prohibiting the use of AA-CHPs would be to change the slope of the time trend; the slope change occurs at most twice (at ${t}_{1}$ and ${t}_{2}$, $1995\le {t}_{1} < {t}_{2}$). The interrupted time-series model we used is presented as follows:

$$\log \,{\mu }_{t}=\,\log \,{m}_{t}+{\rm{\theta }}+{\rm{\phi }}^{\prime} {Z}_{t}+{{\rm{\beta }}}_{0}\,{T}_{t}+{{\rm{\beta }}}_{1}\,{T}_{1t}^{\ast }+{{\rm{\beta }}}_{2}\,{T}_{2t}^{\ast }$$

where ${\mu }_{t}$ is the expected number of new cases, ${m}_{t}$ is the person-years, $t=1995,\ldots ,2013$, ${{\rm{Z}}}_{t}$ is a vector of covariates, ${T}_{t}=t-1994$, ${T}_{1t}^{\ast }=0$ (${\rm{if}}\,t\le {t}_{1}$) and $t-{t}_{1}$ (otherwise), and ${T}_{2t}^{\ast }=0$ (${\rm{if}}\,t\le {t}_{2}$) and $t-{t}_{2}$ (otherwise). The model parameters include ${\rm{\theta }}$, the baseline log incidence rate; ${\rm{\phi }}$, the effects of the covariates; ${{\rm{\beta }}}_{0}$, the baseline slope; ${{\rm{\beta }}}_{1}$, the effect of the slope change at ${t}_{1}$; and ${{\rm{\beta }}}_{2}$, the effect of the slope change at ${t}_{2}$. The slopes after ${t}_{1}$ and ${t}_{2}$ are ${{\rm{\beta }}}_{0}+{{\rm{\beta }}}_{1}$ and ${{\rm{\beta }}}_{0}+{{\rm{\beta }}}_{1}+{{\rm{\beta }}}_{2}$, respectively. We assumed that the number of new cases per year followed a Poisson distribution with mean ${\mu }_{t}$.

We performed model selection based on the AIC (Akaike’s information criterion) index. We specifically considered models with two slope changes (171 models), one slope change (19 models), and no slope change (one model). Subsequently, among all these 191 models, we selected the model with the lowest AIC. We also performed stratified analyses by sex, age (40–59, 60–79, and ≥80 years), region (blackfoot-disease-endemic areas and other areas), and tumor grade (high-grade, low-grade, and unknown grade). For age standardization, we used the World Health Organization (WHO) 2000 standard population (truncated, age ≥40 years).

## Results

**Figure 1:**
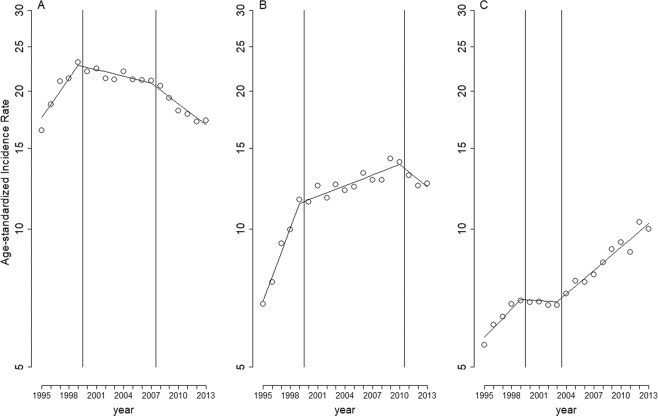
Long-term trends in age-standardized incidence rates per 100,000 population. (**A**) bladder cancer, (**B**) carcinomas of the renal pelvis and other urinary organs, (**C**) kidney cancer. Circles: age-standardized incidence rates per 100,000 population. Solid lines: model estimates. Vertical lines: change-of-slope time points.

Table 1 presents the models with the lowest AIC index for the three cancer types, namely bladder cancer, carcinomas of the renal pelvis and other urinary organs, and kidney cancer. Among the men, the model estimates were higher for bladder cancer (rate ratio [RR] = 2.52) and kidney cancer (RR = 1.92) but lower for carcinomas of the renal pelvis and other urinary organs (RR = 0.9). The model estimates for the three cancer types increased with age but decreased at ages of ≥85 years in all the cancers except for bladder cancer. Figure 1 presents the long-term trends in the age-standardized incidence rate (ASIR) per 100,000 population (circles) and the model estimates (solid line). All three cancer types exhibited two slope changes. In all three cancer types, the first slope change occurred in 2000 (Fig. 1), and all changes were statistically significant (Table 1). The second slope changes occurred in 2008 (for bladder cancer; Fig. 1A), 2011 (for carcinomas of the renal pelvis and other urinary organs; Fig. 1B), and 2004 (for kidney cancer; Fig. 1C); all changes were statistically significant (Table 1).

**Table 1:** Adjusted rate ratios for bladder cancer, carcinomas of the renal pelvis and other urinary organs, and kidney cancer.

	Bladder cancer			Carcinoma of the renal pelvis and other urinary organs			Kidney cancer		
	RR	[95% CI]	P-Value	RR	[95% CI]	P-Value	RR	[95% CI]	P-Value
**Slope**									
baseline	1.07	[1.05–1.08]	<0.001	1.13	[1.11–1.15]	<0.001	1.05	[1.02–1.08]	<0.001
1st slope change	0.92	[0.91–0.94]	<0.001	0.90	[0.88–0.92]	<0.001	0.95	[0.91–0.99]	0.02
2nd slope change	0.98	[0.97–0.99]	<0.001	0.95	[0.93–0.97]	<0.001	1.04	[1.02–1.07]	<0.001
Gender									
Female	1.00		—	1.00		—	1.00		—
Male	2.52	[2.46–2.58]	<0.001	0.90	[0.88–0.93]	<0.001	1.92	[1.85–1.99]	<0.001
Age									
40–44	1.00		—	1.00		—	1.00		—
45–49	2.03	[1.86–2.21]	<0.001	2.04	[1.81–2.30]	<0.001	1.59	[1.45–1.73]	<0.001
50–54	3.70	[3.41–4.00]	<0.001	4.04	[3.62–4.52]	<0.001	2.32	[2.13–2.53]	<0.001
55–59	6.23	[5.76–6.73]	<0.001	7.73	[6.95–8.60]	<0.001	3.22	[2.95–3.50]	<0.001
60–64	10.1	[9.41–10.9]	<0.001	13.2	[11.9–14.6]	<0.001	4.39	[4.04–4.78]	<0.001
65–69	15.3	[14.2–16.5]	<0.001	21.4	[19.3–23.7]	<0.001	5.72	[5.27–6.22]	<0.001
70–74	22.3	[20.7–24.0]	<0.001	27.4	[24.7–30.3]	<0.001	7.08	[6.52–7.69]	<0.001
75–79	30.1	[28.0–32.4]	<0.001	33.1	[29.8–36.6]	<0.001	7.82	[7.18–8.52]	<0.001
80–84	35.9	[33.3–38.7]	<0.001	35.3	[31.7–39.2]	<0.001	8.40	[7.65–9.22]	<0.001
85+	39.8	[36.8–43.1]	<0.001	27.9	[24.9–31.3]	<0.001	7.37	[6.60–8.22]	<0.001

Abbreviations: RR, Rate Ratio; CI, Confidence Interval.

The model-based baseline ASIR increased for the three cancers (7% increase per year, RR = 1.07 for bladder cancer; 13% increase per year, RR = 1.13 for carcinomas of the renal pelvis and other urinary organs; and 5% increase per year, RR = 1.05 for kidney cancer). After the first slope change, the model-based ASIR decreased slightly for bladder cancer (1.6% decrease per year, RR = 1.07 × 0.92 = 0.984), increased slightly for carcinomas of the renal pelvis and other urinary organs (1.7% increase per year, RR = 1.13 × 0.9 = 1.017), and decreased marginally for kidney cancer (0.2% decrease per year, RR = 1.05 × 0.95 = 0.998). After the second slope change, the model-based ASIR decreased considerably for bladder cancer (3.5% decrease per year, RR = 1.07 × 0.92 × 0.98 = 0.965) and carcinomas of the renal pelvis and other urinary organs (3.4% decrease per year, RR = 1.13 × 0.9 × 0.95 = 0.966), but it increased again for kidney cancer (3.7% increase per year, RR = 1.05 × 0.95 × 1.04 = 1.037). The raw data (Supplementary Data, Tables S1–S5) showed that the ASIR reduced about 25% (bladder cancer, from 23.13 per 100,000 population in 1999 to 17.22 per 100,000 population in 2013) and 10% (carcinoma of the renal pelvis and other urinary organs, from 13.98 per 100,000 population in 2010 to 12.55 per 100,000 population in 2013), and increased about 43% (kidney cancer, from 6.98 per 100,000 population in 1999 to 10.00 per 100,000 population in 2013).

**Figure 2:**
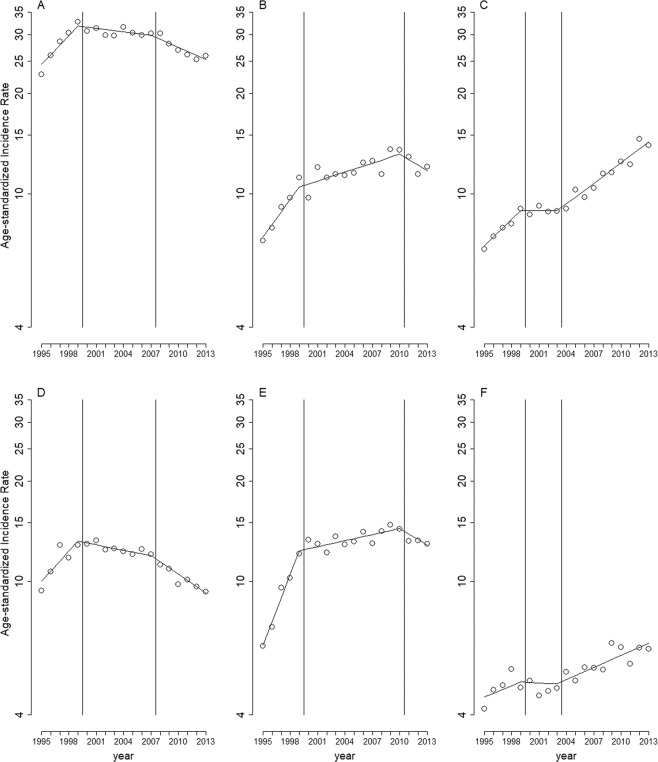
Long-term trends in age-standardized incidence rates per 100,000 population stratified by gender. (**A**) bladder cancer in men, (**B**) carcinomas of the renal pelvis and other urinary organs in men, (**C**) kidney cancer in men, (**D**) bladder cancer in women, (**E**) carcinomas of the renal pelvis and other urinary organs in women, (**F**) kidney cancer in women. Circles: age-standardized incidence rates per 100,000 population. Solid lines: model estimates. Vertical lines: change-of-slope time points.

**Figure 3:**
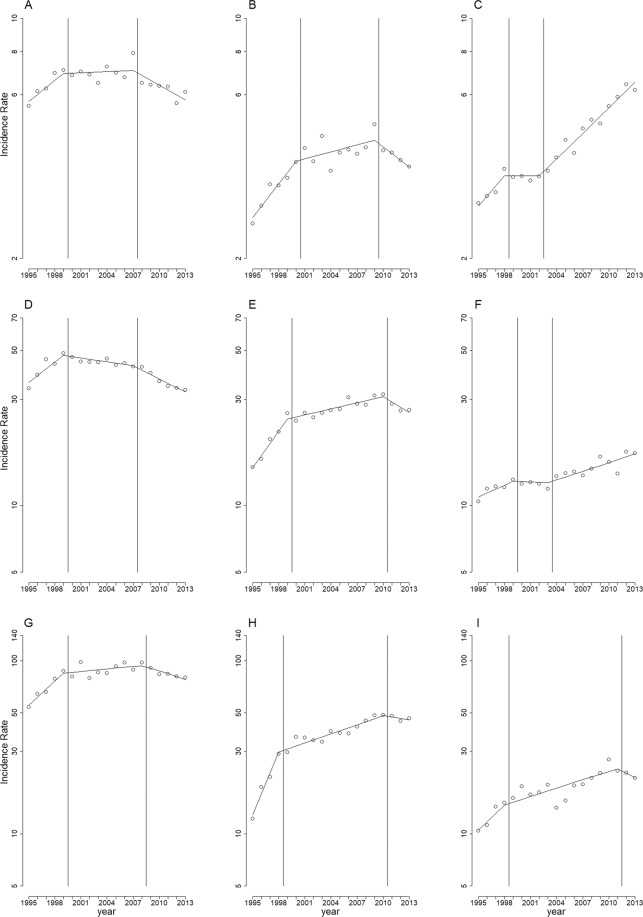
Long-term trends in incidence rates per 100,000 population stratified by age. (**A**) 40–59 years, bladder cancer; (**B**) 40–59 years, carcinomas of the renal pelvis and other urinary organs; (**C**) 40–59 years, kidney cancer; (**D**) 60–79 years, bladder cancer; (**E**) 60–79 years, carcinomas of the renal pelvis and other urinary organs; (**F**) 60–79 years, kidney cancer; (**G**): ≥80 years, bladder cancer; (**H**) ≥80 years, carcinomas of the renal pelvis and other urinary organs; (**I**) ≥80 years, kidney cancer. Circles: age-specific incidence rates per 100,000 population. Solid lines: model estimates. Vertical lines: change-of-slope time points.

**Figure 4:**
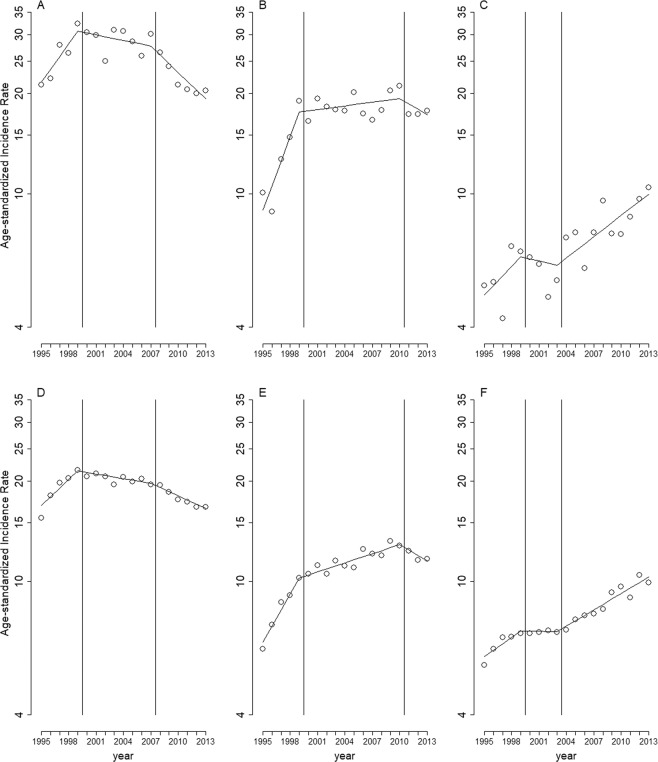
Long-term trends in age-standardized incidence rate per 100,000 population by region. (**A**) bladder cancer in blackfoot-disease-endemic areas, (**B**) carcinomas of the renal pelvis and other urinary organs in blackfoot-disease-endemic areas, (**C**) kidney cancer in blackfoot-disease-endemic areas, (**D**) bladder cancer in other areas, (**E**) carcinomas of the renal pelvis and other urinary organs in other areas, F: kidney cancer in other areas. Circles: age-standardized incidence rates per 100,000 population. Solid lines: model estimates. Vertical lines: change-of-slope time points.

Stratified analyses by sex (Fig. 2), age (Fig. 3), and region (Fig. 4) showed similar long-term trends for the three cancer types as shown in Fig. 1, except for the following minor differences: the first change-of-slope time point occurred one year earlier (Fig. 3C,H,I), and one year later (Fig. 3B), respectively; the second change-of-slope time point occurred one year earlier (Figs. 3B,C, 4C), two years earlier (Fig. 2F), one year later (Fig. 3G), and 8 years later (Fig. 3I), respectively.

We constructed Poisson regression models with an interaction between the second slope change and gender, and the results (Supplementary Data, Table S6) showed that the reduction in incidence rate was greater in female than male for bladder cancer after the second slope change. We also constructed mixed-effects Poisson regression models to adjust for possible spatial heterogeneity (a total of 368 local administrative units in Taiwan), and the results (Supplementary Data, Table S7) were similar to those in Table 1. Autocorrelations and partial autocorrelations of the residuals were examined and found to be nonsignificant (Supplementary Data, Figs. S1–S8).

## Discussion

The common first change-of-slope time point of the three cancer types noted in this study corresponded to the time the first regulatory control of AA-CHPs in Taiwan began (the year 2000). The second change-of-slope time point of the three cancer types all occurred after the year 2003, when a ban on most AA-CHPs and their preparation was imposed in Taiwan. The incidence rates of bladder cancer and carcinomas of the renal pelvis and other urinary organs decreased considerably after 2008 and 2011, respectively. These decreases can be associated with the induction period of these cancer types, which is approximately 10 years long^21^.

The legislation of Tobacco Hazards Prevention Act (THPA) in Taiwan was passed in 1997 and was amended in 2009. Smoking rate in Taiwan in 1995 was high in male (55%) but low in female (3.3%)^28^. The rate decreased to about 45% for male but increased to about 5.5% for female after 1997 and turned to a stable trend after 2001 for both male and female^29^. The rate decreased again after 2009 and the reduction was greater in male (34% in 2013) than female (4.8% in 2013)^29^. We consider the 1997 THPA could be a cause of the first slope change of the three cancer types for male but not for female. We also surmise the 2009 THPA did not have effect on the second slope change of the three cancer types for the following three reasons: (i) the change-of-slope time points for bladder cancer (2000 and 2008) were all before 2009, (ii) the effect of 2009 THPA should be greater in male than female, but the interaction between the second slope change and gender implied otherwise for bladder cancer and was not significant for carcinomas of the renal pelvis and other urinary organs (Supplementary Data, Table S6), and (iii) the implementation of 2009 THPA may not affect the incidence of urological cancers because slope changes for kidney cancer did not occur after 2009.

Between 1997 and 2003 in Taiwan, the prescription rate of AA-CHPs was 31.6 per 1000 person-years for female and 25.9 per 1000 person-years for male^30^. This helps to explain why the reduction of incidence rate was greater in female than male after the second slope change for bladder cancer. Stratified analyses by grade for bladder cancer and carcinoma of the renal pelvis and other urinary organs (Supplementary Data, Fig. S9) showed that the incidence rates decreased for low-grade tumors and tumors with unknown grade but increased for high-grade tumors. However, after the second slope changes for the two cancer types (2008 and 2011, respectively), the incidence rate decreased considerably for low-grade tumors and tumors with unknown grade and the increasing trend was leveling off for high-grade tumors.

The Multiple Primary and Histology Coding Rules manual published by the Surveillance, Epidemiology, and End Results has been used since 2007^31^. Only the cancer type with the earliest occurrence is registered if a patient simultaneously exhibits at least two cancer types all having morphology from urothelial carcinoma. This may have caused an underestimation of the incidence of bladder cancer and carcinomas of the renal pelvis and other urinary organs. Furthermore, data regarding some potential confounding factors, such as stage, alcohol consumption, obesity, hypertension, red and processed meat intake, occupational exposure, and genetic susceptibility^21,32–36^, were not collected in this study. Additional studies are required to examine whether these risk factors have changed over time, and if affirmative, they may also contribute to the decline of the incidence rates for the two cancer types.

In summary, this investigation was a national population-based study. We found that the reduction of the incidence rate of bladder cancer and carcinomas of the renal pelvis and other urinary organs was possibly associated with the cessation of AA-CHPs in Taiwan. Further studies are warranted to examine whether other risk factors contribute to the decline of the incidence rates for the two cancer types.

## Supplementary information

Supplementary Information
