# Supplementary material for: Reduction in the Incidence of Urological Cancers after the Ban on Chinese Herbal Products Containing Aristolochic Acid: An Interrupted Time-Series Analysis
Source: Sci Rep. 2019 Dec 27;9:19860. doi: 10.1038/s41598-019-56394-y (PMC6934535; doi:10.1038/s41598-019-56394-y)
Supplement: Supplementary file 1 — Supplementary Information [file 41598_2019_56394_MOESM1_ESM.doc]

**Title**: Reduction in the Incidence of Urological Cancers after the Ban on Chinese Herbal Products Containing Aristolochic Acid: An Interrupted Time-Series Analysis

**Authors**: Jing-Rong Jhuang,^1,2^ Chun-Ju Chiang,^1,2^ Shih-Yung Su,^1,3^ Ya-Wen Yang^2^, Wen-Chung Lee ^1,2,3*^

**Authors’ affiliations**:

^1^Institute of Epidemiology and Preventive Medicine, College of Public Health, National Taiwan University, Taipei, Taiwan

^2^Taiwan Cancer Registry, Taipei, Taiwan

^3^Innovation and Policy Center for Population Health and Sustainable Environment, College of Public Health, National Taiwan University, Taipei, Taiwan

***Corresponding author**:

Professor Wen-Chung Lee, Institute of Epidemiology and Preventive Medicine, College of Public Health, National Taiwan University, Rm. 536, No. 17, Xuzhou Rd., Taipei 100, Taiwan. E-mail: wenchung@ntu.edu.tw

**Table S1.** Number of cases, crude incidence rate per 100,000 population, and age-standardized incidence rate per 100,000 population for bladder cancer, carcinoma of the renal pelvis and other urinary organs, and kidney cancer. Abbreviations: N, Number of cases; CIR, Crude Incidence Rate; ASIR, Age-standardized Incidence Rate.

|  |  | **Bladder cancer** | | |  | **Carcinoma of the renal pelvis and other urinary organs** | | |  | **Kidney cancer** | | |
| --- | --- | --- | --- | --- | --- | --- | --- | --- | --- | --- | --- | --- |
| **Year** |  | **N** | **CIR** | **ASIR** |  | **N** | **CIR** | **ASIR** |  | **N** | **CIR** | **ASIR** |
| 1995 |  | 1066 | 15.97 | 16.39 |  | 448 | 6.71 | 6.85 |  | 367 | 5.50 | 5.57 |
| 1996 |  | 1258 | 18.19 | 18.71 |  | 518 | 7.49 | 7.66 |  | 418 | 6.04 | 6.17 |
| 1997 |  | 1455 | 20.32 | 21.00 |  | 642 | 8.97 | 9.30 |  | 449 | 6.27 | 6.42 |
| 1998 |  | 1520 | 20.49 | 21.29 |  | 717 | 9.66 | 9.96 |  | 489 | 6.59 | 6.85 |
| 1999 |  | 1716 | 22.32 | 23.13 |  | 854 | 11.11 | 11.57 |  | 520 | 6.76 | 6.98 |
| 2000 |  | 1700 | 21.37 | 22.07 |  | 875 | 11.00 | 11.45 |  | 534 | 6.71 | 6.91 |
| 2001 |  | 1762 | 21.43 | 22.39 |  | 973 | 11.84 | 12.43 |  | 544 | 6.62 | 6.93 |
| 2002 |  | 1749 | 20.61 | 21.29 |  | 948 | 11.17 | 11.70 |  | 563 | 6.64 | 6.82 |
| 2003 |  | 1795 | 20.51 | 21.17 |  | 1043 | 11.92 | 12.50 |  | 579 | 6.62 | 6.82 |
| 2004 |  | 1938 | 21.49 | 22.04 |  | 1054 | 11.69 | 12.14 |  | 637 | 7.06 | 7.22 |
| 2005 |  | 1924 | 20.74 | 21.18 |  | 1111 | 11.98 | 12.34 |  | 701 | 7.56 | 7.69 |
| 2006 |  | 2009 | 21.06 | 21.10 |  | 1231 | 12.90 | 13.23 |  | 715 | 7.50 | 7.64 |
| 2007 |  | 2065 | 21.09 | 21.03 |  | 1218 | 12.44 | 12.78 |  | 769 | 7.86 | 7.95 |
| 2008 |  | 2073 | 20.68 | 20.49 |  | 1278 | 12.75 | 12.80 |  | 844 | 8.42 | 8.45 |
| 2009 |  | 2035 | 19.81 | 19.29 |  | 1468 | 14.29 | 14.24 |  | 924 | 9.00 | 9.02 |
| 2010 |  | 1976 | 18.79 | 18.12 |  | 1479 | 14.06 | 13.98 |  | 997 | 9.48 | 9.35 |
| 2011 |  | 2004 | 18.65 | 17.80 |  | 1444 | 13.44 | 13.08 |  | 980 | 9.12 | 8.89 |
| 2012 |  | 1996 | 18.20 | 17.15 |  | 1407 | 12.83 | 12.42 |  | 1156 | 10.54 | 10.34 |
| 2013 |  | 2070 | 18.52 | 17.22 |  | 1469 | 13.14 | 12.55 |  | 1153 | 10.32 | 10.00 |

**Table S2.** Number of cases, crude incidence rate per 100,000 population, and age-standardized incidence rate per 100,000 population stratified by gender for bladder cancer, carcinoma of the renal pelvis and other urinary organs, and kidney cancer.

|  |  | **Bladder cancer** | | |  | **Carcinoma of the renal pelvis and other urinary organs** | | |  | **Kidney cancer** | | |
| --- | --- | --- | --- | --- | --- | --- | --- | --- | --- | --- | --- | --- |
| **Year** |  | **N** | **CIR** | **ASIR** |  | **N** | **CIR** | **ASIR** |  | **N** | **CIR** | **ASIR** |
| **Male** |  |  |  |  |  |  |  |  |  |  |  |  |
| 1995 |  | 780 | 22.61 | 22.81 |  | 253 | 7.33 | 7.24 |  | 238 | 6.90 | 6.84 |
| 1996 |  | 921 | 25.86 | 26.02 |  | 287 | 8.06 | 7.92 |  | 267 | 7.50 | 7.46 |
| 1997 |  | 1033 | 28.12 | 28.52 |  | 328 | 8.93 | 9.14 |  | 284 | 7.73 | 7.92 |
| 1998 |  | 1119 | 29.50 | 30.32 |  | 366 | 9.65 | 9.73 |  | 302 | 7.96 | 8.15 |
| 1999 |  | 1260 | 32.17 | 32.66 |  | 425 | 10.85 | 11.17 |  | 347 | 8.86 | 9.04 |
| 2000 |  | 1220 | 30.20 | 30.70 |  | 382 | 9.46 | 9.74 |  | 345 | 8.54 | 8.69 |
| 2001 |  | 1253 | 30.11 | 31.26 |  | 478 | 11.49 | 12.04 |  | 371 | 8.92 | 9.23 |
| 2002 |  | 1250 | 29.19 | 29.83 |  | 465 | 10.86 | 11.19 |  | 374 | 8.73 | 8.84 |
| 2003 |  | 1270 | 28.83 | 29.67 |  | 483 | 10.96 | 11.45 |  | 374 | 8.49 | 8.87 |
| 2004 |  | 1405 | 31.05 | 31.61 |  | 500 | 11.05 | 11.37 |  | 406 | 8.97 | 9.01 |
| 2005 |  | 1384 | 29.82 | 30.29 |  | 519 | 11.18 | 11.57 |  | 471 | 10.15 | 10.29 |
| 2006 |  | 1420 | 29.84 | 29.80 |  | 576 | 12.11 | 12.39 |  | 453 | 9.52 | 9.79 |
| 2007 |  | 1468 | 30.16 | 30.16 |  | 590 | 12.12 | 12.56 |  | 504 | 10.35 | 10.43 |
| 2008 |  | 1488 | 29.94 | 30.21 |  | 559 | 11.25 | 11.44 |  | 571 | 11.49 | 11.52 |
| 2009 |  | 1443 | 28.43 | 28.08 |  | 686 | 13.51 | 13.62 |  | 582 | 11.47 | 11.61 |
| 2010 |  | 1428 | 27.57 | 26.94 |  | 688 | 13.28 | 13.55 |  | 650 | 12.55 | 12.51 |
| 2011 |  | 1418 | 26.86 | 26.04 |  | 679 | 12.86 | 12.88 |  | 657 | 12.45 | 12.27 |
| 2012 |  | 1413 | 26.32 | 25.29 |  | 626 | 11.66 | 11.47 |  | 788 | 14.68 | 14.58 |
| 2013 |  | 1485 | 27.22 | 25.89 |  | 670 | 12.28 | 12.05 |  | 774 | 14.19 | 13.98 |
| **Female** |  |  |  |  |  |  |  |  |  |  |  |  |
| 1995 |  | 286 | 8.87 | 9.39 |  | 195 | 6.05 | 6.41 |  | 129 | 4.00 | 4.16 |
| 1996 |  | 337 | 10.05 | 10.72 |  | 231 | 6.89 | 7.32 |  | 151 | 4.50 | 4.74 |
| 1997 |  | 422 | 12.11 | 12.87 |  | 314 | 9.01 | 9.57 |  | 165 | 4.73 | 4.90 |
| 1998 |  | 401 | 11.06 | 11.77 |  | 351 | 9.68 | 10.27 |  | 187 | 5.16 | 5.48 |
| 1999 |  | 456 | 12.09 | 12.87 |  | 429 | 11.38 | 12.09 |  | 173 | 4.59 | 4.82 |
| 2000 |  | 480 | 12.26 | 12.98 |  | 493 | 12.59 | 13.30 |  | 189 | 4.83 | 5.05 |
| 2001 |  | 509 | 12.54 | 13.27 |  | 495 | 12.20 | 12.95 |  | 173 | 4.26 | 4.55 |
| 2002 |  | 499 | 11.87 | 12.46 |  | 483 | 11.49 | 12.23 |  | 189 | 4.50 | 4.71 |
| 2003 |  | 525 | 12.08 | 12.58 |  | 560 | 12.88 | 13.66 |  | 205 | 4.72 | 4.81 |
| 2004 |  | 533 | 11.87 | 12.31 |  | 554 | 12.34 | 12.92 |  | 231 | 5.14 | 5.37 |
| 2005 |  | 540 | 11.65 | 12.06 |  | 592 | 12.78 | 13.16 |  | 230 | 4.96 | 5.05 |
| 2006 |  | 589 | 12.32 | 12.50 |  | 655 | 13.70 | 14.06 |  | 262 | 5.48 | 5.54 |
| 2007 |  | 597 | 12.13 | 12.08 |  | 628 | 12.76 | 13.03 |  | 265 | 5.38 | 5.52 |
| 2008 |  | 585 | 11.57 | 11.25 |  | 719 | 14.22 | 14.14 |  | 273 | 5.40 | 5.45 |
| 2009 |  | 592 | 11.39 | 10.94 |  | 782 | 15.05 | 14.80 |  | 342 | 6.58 | 6.54 |
| 2010 |  | 548 | 10.27 | 9.83 |  | 791 | 14.82 | 14.38 |  | 347 | 6.50 | 6.37 |
| 2011 |  | 586 | 10.71 | 10.13 |  | 765 | 13.99 | 13.22 |  | 323 | 5.91 | 5.67 |
| 2012 |  | 583 | 10.42 | 9.67 |  | 781 | 13.95 | 13.25 |  | 368 | 6.58 | 6.34 |
| 2013 |  | 585 | 10.23 | 9.31 |  | 799 | 13.97 | 12.97 |  | 379 | 6.63 | 6.30 |

Abbreviations: N, Number of cases; CIR, Crude Incidence Rate; ASIR, Age-standardized Incidence Rate.

**Table S3.** Number of cases and crude incidence rate per 100,000 population stratified by age for bladder cancer, carcinoma of the renal pelvis and other urinary organs, and kidney cancer. Abbreviations: N, Number of cases; CIR, Crude Incidence Rate.

|  |  | **Bladder cancer** | | |  | **Carcinoma of the renal pelvis and other urinary organs** | | |  | **Kidney cancer** | | |
| --- | --- | --- | --- | --- | --- | --- | --- | --- | --- | --- | --- | --- |
| **Year** |  | **N** |  | **CIR** |  | **N** |  | **CIR** |  | **N** |  | **CIR** |
| **40-59 years** | |  |  |  |  |  |  |  |  |  |  |  |
| 1995 |  | 242 |  | 5.55 |  | 110 |  | 2.52 |  | 126 |  | 2.89 |
| 1996 |  | 279 |  | 6.14 |  | 129 |  | 2.84 |  | 138 |  | 3.04 |
| 1997 |  | 295 |  | 6.24 |  | 155 |  | 3.28 |  | 147 |  | 3.11 |
| 1998 |  | 340 |  | 6.92 |  | 160 |  | 3.26 |  | 179 |  | 3.64 |
| 1999 |  | 361 |  | 7.06 |  | 175 |  | 3.42 |  | 176 |  | 3.44 |
| 2000 |  | 362 |  | 6.83 |  | 202 |  | 3.81 |  | 184 |  | 3.47 |
| 2001 |  | 384 |  | 6.99 |  | 230 |  | 4.19 |  | 185 |  | 3.37 |
| 2002 |  | 390 |  | 6.86 |  | 218 |  | 3.83 |  | 197 |  | 3.46 |
| 2003 |  | 382 |  | 6.49 |  | 267 |  | 4.54 |  | 212 |  | 3.60 |
| 2004 |  | 440 |  | 7.23 |  | 219 |  | 3.60 |  | 239 |  | 3.93 |
| 2005 |  | 437 |  | 6.94 |  | 256 |  | 4.07 |  | 279 |  | 4.43 |
| 2006 |  | 439 |  | 6.74 |  | 270 |  | 4.14 |  | 264 |  | 4.05 |
| 2007 |  | 531 |  | 7.93 |  | 270 |  | 4.03 |  | 320 |  | 4.78 |
| 2008 |  | 444 |  | 6.49 |  | 288 |  | 4.21 |  | 346 |  | 5.06 |
| 2009 |  | 446 |  | 6.40 |  | 342 |  | 4.91 |  | 344 |  | 4.94 |
| 2010 |  | 450 |  | 6.36 |  | 292 |  | 4.13 |  | 392 |  | 5.54 |
| 2011 |  | 451 |  | 6.32 |  | 290 |  | 4.06 |  | 422 |  | 5.91 |
| 2012 |  | 406 |  | 5.66 |  | 277 |  | 3.86 |  | 461 |  | 6.43 |
| 2013 |  | 439 |  | 6.10 |  | 266 |  | 3.69 |  | 445 |  | 6.18 |
| **60-79 years** | |  |  |  |  |  |  |  |  |  |  |  |
| 1995 |  | 705 |  | 33.65 |  | 311 |  | 14.85 |  | 218 |  | 10.41 |
| 1996 |  | 830 |  | 38.74 |  | 346 |  | 16.15 |  | 254 |  | 11.86 |
| 1997 |  | 999 |  | 45.57 |  | 435 |  | 19.84 |  | 267 |  | 12.18 |
| 1998 |  | 977 |  | 43.47 |  | 482 |  | 21.45 |  | 271 |  | 12.06 |
| 1999 |  | 1115 |  | 48.41 |  | 598 |  | 25.96 |  | 300 |  | 13.03 |
| 2000 |  | 1101 |  | 46.66 |  | 567 |  | 24.03 |  | 295 |  | 12.50 |
| 2001 |  | 1070 |  | 44.37 |  | 630 |  | 26.12 |  | 306 |  | 12.69 |
| 2002 |  | 1088 |  | 44.29 |  | 612 |  | 24.91 |  | 307 |  | 12.50 |
| 2003 |  | 1101 |  | 44.04 |  | 652 |  | 26.08 |  | 297 |  | 11.88 |
| 2004 |  | 1167 |  | 45.94 |  | 682 |  | 26.85 |  | 343 |  | 13.50 |
| 2005 |  | 1098 |  | 42.88 |  | 695 |  | 27.14 |  | 357 |  | 13.94 |
| 2006 |  | 1127 |  | 43.82 |  | 789 |  | 30.68 |  | 365 |  | 14.19 |
| 2007 |  | 1101 |  | 42.25 |  | 746 |  | 28.63 |  | 355 |  | 13.62 |
| 2008 |  | 1123 |  | 42.13 |  | 756 |  | 28.36 |  | 389 |  | 14.59 |
| 2009 |  | 1086 |  | 39.50 |  | 859 |  | 31.24 |  | 456 |  | 16.58 |
| 2010 |  | 1035 |  | 36.26 |  | 902 |  | 31.60 |  | 447 |  | 15.66 |
| 2011 |  | 1031 |  | 34.49 |  | 856 |  | 28.63 |  | 415 |  | 13.88 |
| 2012 |  | 1065 |  | 33.85 |  | 840 |  | 26.69 |  | 549 |  | 17.45 |
| 2013 |  | 1096 |  | 33.16 |  | 891 |  | 26.96 |  | 567 |  | 17.16 |
| **>=80 years** |  |  |  |  |  |  |  |  |  |  |  |  |
| 1995 |  | 119 |  | 53.97 |  | 27 |  | 12.24 |  | 23 |  | 10.43 |
| 1996 |  | 149 |  | 64.43 |  | 43 |  | 18.59 |  | 26 |  | 11.24 |
| 1997 |  | 161 |  | 66.12 |  | 52 |  | 21.35 |  | 35 |  | 14.37 |
| 1998 |  | 203 |  | 78.58 |  | 75 |  | 29.03 |  | 39 |  | 15.10 |
| 1999 |  | 240 |  | 87.66 |  | 81 |  | 29.59 |  | 44 |  | 16.07 |
| 2000 |  | 237 |  | 81.28 |  | 106 |  | 36.35 |  | 55 |  | 18.86 |
| 2001 |  | 308 |  | 97.96 |  | 113 |  | 35.94 |  | 53 |  | 16.86 |
| 2002 |  | 271 |  | 79.67 |  | 118 |  | 34.69 |  | 59 |  | 17.35 |
| 2003 |  | 312 |  | 85.52 |  | 124 |  | 33.99 |  | 70 |  | 19.19 |
| 2004 |  | 331 |  | 84.89 |  | 153 |  | 39.24 |  | 55 |  | 14.11 |
| 2005 |  | 389 |  | 92.87 |  | 160 |  | 38.20 |  | 65 |  | 15.52 |
| 2006 |  | 443 |  | 97.76 |  | 172 |  | 37.96 |  | 86 |  | 18.98 |
| 2007 |  | 433 |  | 88.97 |  | 202 |  | 41.51 |  | 94 |  | 19.32 |
| 2008 |  | 506 |  | 97.60 |  | 234 |  | 45.13 |  | 109 |  | 21.02 |
| 2009 |  | 503 |  | 91.02 |  | 267 |  | 48.31 |  | 124 |  | 22.44 |
| 2010 |  | 491 |  | 83.58 |  | 285 |  | 48.51 |  | 158 |  | 26.89 |
| 2011 |  | 522 |  | 84.21 |  | 298 |  | 48.08 |  | 143 |  | 23.07 |
| 2012 |  | 525 |  | 81.10 |  | 290 |  | 44.80 |  | 146 |  | 22.55 |
| 2013 |  | 535 |  | 79.70 |  | 312 |  | 46.48 |  | 141 |  | 21.01 |

|  |  | **Bladder cancer** | | |  | **Carcinoma of the renal pelvis and other urinary organs** | | |  | **Kidney cancer** | | |
| --- | --- | --- | --- | --- | --- | --- | --- | --- | --- | --- | --- | --- |
| **Year** |  | **N** | **CIR** | **ASIR** |  | **N** | **CIR** | **ASIR** |  | **N** | **CIR** | **ASIR** |
| **BDE areas** | |  |  |  |  |  |  |  |  |  |  |  |
| 1995 |  | 210 | 21.58 | 21.24 |  | 101 | 10.38 | 10.08 |  | 52 | 5.34 | 5.31 |
| 1996 |  | 225 | 22.40 | 22.17 |  | 92 | 9.16 | 8.85 |  | 56 | 5.58 | 5.46 |
| 1997 |  | 297 | 28.66 | 27.90 |  | 136 | 13.12 | 12.70 |  | 46 | 4.44 | 4.25 |
| 1998 |  | 279 | 26.12 | 26.34 |  | 161 | 15.07 | 14.76 |  | 71 | 6.65 | 6.99 |
| 1999 |  | 358 | 32.51 | 32.26 |  | 212 | 19.25 | 19.01 |  | 76 | 6.90 | 6.74 |
| 2000 |  | 350 | 30.84 | 30.46 |  | 190 | 16.74 | 16.52 |  | 73 | 6.43 | 6.48 |
| 2001 |  | 354 | 30.31 | 29.88 |  | 231 | 19.78 | 19.26 |  | 74 | 6.34 | 6.18 |
| 2002 |  | 300 | 24.98 | 24.93 |  | 220 | 18.32 | 18.23 |  | 61 | 5.08 | 4.93 |
| 2003 |  | 385 | 31.19 | 30.88 |  | 222 | 17.99 | 17.89 |  | 68 | 5.51 | 5.51 |
| 2004 |  | 395 | 31.18 | 30.66 |  | 232 | 18.32 | 17.76 |  | 97 | 7.66 | 7.40 |
| 2005 |  | 378 | 29.13 | 28.53 |  | 265 | 20.42 | 20.10 |  | 101 | 7.78 | 7.68 |
| 2006 |  | 356 | 26.80 | 25.90 |  | 243 | 18.30 | 17.39 |  | 81 | 6.10 | 5.99 |
| 2007 |  | 421 | 31.03 | 30.12 |  | 232 | 17.10 | 16.65 |  | 105 | 7.74 | 7.66 |
| 2008 |  | 391 | 28.24 | 26.53 |  | 254 | 18.34 | 17.77 |  | 137 | 9.89 | 9.56 |
| 2009 |  | 365 | 25.81 | 24.05 |  | 304 | 21.49 | 20.41 |  | 112 | 7.92 | 7.61 |
| 2010 |  | 336 | 23.28 | 21.23 |  | 323 | 22.38 | 21.01 |  | 115 | 7.97 | 7.57 |
| 2011 |  | 332 | 22.58 | 20.53 |  | 274 | 18.63 | 17.35 |  | 134 | 9.11 | 8.55 |
| 2012 |  | 332 | 22.19 | 19.95 |  | 283 | 18.91 | 17.35 |  | 154 | 10.29 | 9.67 |
| 2013 |  | 347 | 22.81 | 20.37 |  | 293 | 19.26 | 17.75 |  | 166 | 10.91 | 10.46 |
| **Other areas** | |  |  |  |  |  |  |  |  |  |  |  |
| 1995 |  | 856 | 15.01 | 15.52 |  | 347 | 6.09 | 6.28 |  | 315 | 5.53 | 5.63 |
| 1996 |  | 1033 | 17.47 | 18.06 |  | 426 | 7.21 | 7.43 |  | 362 | 6.12 | 6.29 |
| 1997 |  | 1158 | 18.91 | 19.75 |  | 506 | 8.26 | 8.67 |  | 403 | 6.58 | 6.81 |
| 1998 |  | 1241 | 19.54 | 20.42 |  | 556 | 8.75 | 9.09 |  | 418 | 6.58 | 6.84 |
| 1999 |  | 1358 | 20.62 | 21.51 |  | 642 | 9.75 | 10.24 |  | 444 | 6.74 | 7.01 |
| 2000 |  | 1350 | 19.79 | 20.61 |  | 685 | 10.04 | 10.55 |  | 461 | 6.76 | 6.99 |
| 2001 |  | 1408 | 19.97 | 21.03 |  | 742 | 10.52 | 11.20 |  | 470 | 6.66 | 7.06 |
| 2002 |  | 1449 | 19.89 | 20.66 |  | 728 | 9.99 | 10.55 |  | 502 | 6.89 | 7.15 |
| 2003 |  | 1410 | 18.76 | 19.49 |  | 821 | 10.92 | 11.57 |  | 511 | 6.80 | 7.05 |
| 2004 |  | 1543 | 19.91 | 20.57 |  | 822 | 10.61 | 11.13 |  | 540 | 6.97 | 7.18 |
| 2005 |  | 1546 | 19.38 | 19.94 |  | 846 | 10.60 | 11.00 |  | 600 | 7.52 | 7.69 |
| 2006 |  | 1653 | 20.13 | 20.30 |  | 988 | 12.03 | 12.51 |  | 634 | 7.72 | 7.93 |
| 2007 |  | 1644 | 19.50 | 19.51 |  | 986 | 11.69 | 12.12 |  | 664 | 7.87 | 8.01 |
| 2008 |  | 1682 | 19.47 | 19.46 |  | 1024 | 11.85 | 11.96 |  | 707 | 8.18 | 8.26 |
| 2009 |  | 1670 | 18.85 | 18.50 |  | 1164 | 13.14 | 13.19 |  | 812 | 9.17 | 9.27 |
| 2010 |  | 1640 | 18.08 | 17.60 |  | 1156 | 12.74 | 12.79 |  | 882 | 9.72 | 9.64 |
| 2011 |  | 1672 | 18.02 | 17.34 |  | 1170 | 12.61 | 12.36 |  | 846 | 9.12 | 8.94 |
| 2012 |  | 1664 | 17.57 | 16.69 |  | 1124 | 11.87 | 11.59 |  | 1002 | 10.58 | 10.44 |
| 2013 |  | 1723 | 17.85 | 16.70 |  | 1176 | 12.18 | 11.68 |  | 987 | 10.22 | 9.94 |

**Table S4.** Number of cases, crude incidence rate per 100,000 population, and age-standardized incidence rate per 100,000 population stratified by region for bladder cancer, carcinoma of the renal pelvis and other urinary organs, and kidney cancer. Abbreviations: N, Number of cases; CIR, Crude Incidence Rate; ASIR, Age-standardized Incidence Rate; BDE, blackfoot-disease endemic.

**Table S5.** Number of cases, crude incidence rate per 100,000 population, and age-standardized incidence rate per 100,000 population stratified by grade for bladder cancer and carcinoma of the renal pelvis and other urinary organs. Abbreviations: N, Number of cases; CIR, Crude Incidence Rate; ASIR, Age-standardized Incidence Rate.

|  |  | **Bladder cancer** | | |  | **Carcinoma of the renal pelvis and other urinary organs** | | |
| --- | --- | --- | --- | --- | --- | --- | --- | --- |
| **Year** |  | **N** | **CIR** | **ASIR** |  | **N** | **CIR** | **ASIR** |
| **High-grade** | |  |  |  |  |  |  |  |
| 2004 |  | 823 | 9.13 | 9.39 |  | 465 | 5.16 | 5.38 |
| 2005 |  | 931 | 10.04 | 10.26 |  | 619 | 6.67 | 6.91 |
| 2006 |  | 1051 | 11.02 | 11.05 |  | 695 | 7.29 | 7.49 |
| 2007 |  | 1122 | 11.46 | 11.47 |  | 729 | 7.45 | 7.68 |
| 2008 |  | 1167 | 11.64 | 11.51 |  | 788 | 7.86 | 7.94 |
| 2009 |  | 1217 | 11.85 | 11.57 |  | 909 | 8.85 | 8.81 |
| 2010 |  | 1253 | 11.92 | 11.55 |  | 963 | 9.16 | 9.20 |
| 2011 |  | 1387 | 12.90 | 12.32 |  | 1013 | 9.43 | 9.27 |
| 2012 |  | 1407 | 12.83 | 12.09 |  | 997 | 9.09 | 8.88 |
| 2013 |  | 1522 | 13.62 | 12.64 |  | 1074 | 9.61 | 9.26 |
| **Low-grade** | |  |  |  |  |  |  |  |
| 2004 |  | 813 | 9.20 | 9.02 |  | 339 | 3.76 | 3.92 |
| 2005 |  | 718 | 7.92 | 7.74 |  | 270 | 2.91 | 3.00 |
| 2006 |  | 646 | 6.83 | 6.77 |  | 260 | 2.73 | 2.78 |
| 2007 |  | 637 | 6.44 | 6.51 |  | 239 | 2.44 | 2.52 |
| 2008 |  | 577 | 5.75 | 5.76 |  | 255 | 2.54 | 2.56 |
| 2009 |  | 542 | 5.13 | 5.28 |  | 270 | 2.63 | 2.66 |
| 2010 |  | 423 | 3.88 | 4.02 |  | 214 | 2.04 | 2.03 |
| 2011 |  | 380 | 3.38 | 3.54 |  | 180 | 1.67 | 1.62 |
| 2012 |  | 405 | 3.48 | 3.69 |  | 179 | 1.63 | 1.59 |
| 2013 |  | 368 | 3.14 | 3.29 |  | 140 | 1.25 | 1.21 |
| **Unknown grade** | |  |  |  |  |  |  |  |
| 2004 |  | 302 | 3.35 | 3.45 |  | 250 | 2.77 | 2.84 |
| 2005 |  | 275 | 2.96 | 3.00 |  | 222 | 2.39 | 2.43 |
| 2006 |  | 312 | 3.27 | 3.22 |  | 276 | 2.89 | 2.96 |
| 2007 |  | 306 | 3.13 | 3.12 |  | 250 | 2.55 | 2.58 |
| 2008 |  | 329 | 3.28 | 3.23 |  | 235 | 2.34 | 2.30 |
| 2009 |  | 276 | 2.69 | 2.59 |  | 289 | 2.81 | 2.77 |
| 2010 |  | 300 | 2.85 | 2.69 |  | 302 | 2.87 | 2.75 |
| 2011 |  | 237 | 2.21 | 2.10 |  | 251 | 2.34 | 2.18 |
| 2012 |  | 184 | 1.68 | 1.57 |  | 231 | 2.11 | 1.95 |
| 2013 |  | 180 | 1.61 | 1.43 |  | 255 | 2.28 | 2.09 |

**Table S6.** Adjusted rate ratios for bladder cancer, carcinoma of the renal pelvis and other urinary organs, and kidney cancer (Poisson regression models with interaction). Abbreviations: RR, Rate Ratio; CI, Confidence Interval.

|  | **Bladder cancer** | | |  | **Carcinoma of the renal pelvis and other urinary organs** | | |
| --- | --- | --- | --- | --- | --- | --- | --- |
|  | **RR** | **[95% CI]** | **P-Value** |  | **RR** | **[95% CI]** | **P-Value** |
| Slope |  |  |  |  |  |  |  |
| baseline | 1.07 | [1.05–1.08] | <0.001 |  | 1.13 | [1.11–1.15] | <0.001 |
| 1st slope change | 0.92 | [0.91–0.94] | <0.001 |  | 0.90 | [0.88–0.92] | <0.001 |
| 2nd slope change | 0.98 | [0.97–0.99] | 0.002 |  | 0.95 | [0.92–0.97] | <0.001 |
| Gender |  |  |  |  |  |  |  |
| Female | 1.00 | **–** | **–** |  | 1.00 | **–** | **–** |
| Male | 2.46 | [2.39–2.53] | <0.001 |  | 0.90 | [0.87–0.93] | <0.001 |
| Age |  |  |  |  |  |  |  |
| 40-44 | 1.00 | **–** | **–** |  | 1.00 | **–** | **–** |
| 45-49 | 2.03 | [1.86–2.21] | <0.001 |  | 2.04 | [1.81–2.30] | <0.001 |
| 50-54 | 3.70 | [3.41–4.00] | <0.001 |  | 4.04 | [3.62–4.52] | <0.001 |
| 55-59 | 6.23 | [5.76–6.73] | <0.001 |  | 7.73 | [6.95–8.60] | <0.001 |
| 60-64 | 10.2 | [9.40–10.9] | <0.001 |  | 13.2 | [11.9–14.6] | <0.001 |
| 65-69 | 15.3 | [14.2–16.5] | <0.001 |  | 21.4 | [19.3–23.7] | <0.001 |
| 70-74 | 22.3 | [20.7–24.0] | <0.001 |  | 27.4 | [24.7–30.3] | <0.001 |
| 75-79 | 30.2 | [28.0–32.4] | <0.001 |  | 33.1 | [29.8–36.6] | <0.001 |
| 80-84 | 35.9 | [33.3–38.7] | <0.001 |  | 35.3 | [31.7–39.2] | <0.001 |
| 85+ | 39.8 | [36.8–43.1] | <0.001 |  | 27.9 | [24.9–31.3] | <0.001 |
| Interaction |  |  |  |  |  |  |  |
| Female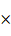2nd slope change | 1.00 | **–** | **–** |  | 1.00 | **–** | **–** |
| Male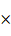2nd slope change | 1.02 | [1.01–1.03] | 0.002 |  | 1.00 | [0.97–1.04] | 0.783 |

**Table S7.** Adjusted rate ratios for bladder cancer, carcinoma of the renal pelvis and other urinary organs, and kidney cancer (mixed-effects Poisson regression models). Abbreviations: RR, Rate Ratio; CI, Confidence Interval.

|  | **Bladder cancer** | | |  | **Carcinoma of the renal pelvis and other urinary organs** | | |  | **Kidney cancer** | | |
| --- | --- | --- | --- | --- | --- | --- | --- | --- | --- | --- | --- |
|  | **RR** | **[95% CI]** | **P-Value** |  | **RR** | **[95% CI]** | **P-Value** |  | **RR** | **[95% CI]** | **P-Value** |
| Slope |  |  |  |  |  |  |  |  |  |  |  |
| baseline | 1.07 | [1.06–1.09] | <0.001 |  | 1.13 | [1.11–1.15] | <0.001 |  | 1.05 | [1.02–1.08] | <0.001 |
| 1st slope change | 0.92 | [0.91–0.94] | <0.001 |  | 0.90 | [0.88–0.92] | <0.001 |  | 0.95 | [0.92–0.99] | 0.02 |
| 2nd slope change | 0.98 | [0.97–0.99] | <0.001 |  | 0.95 | [0.93–0.97] | <0.001 |  | 1.04 | [1.02–1.07] | <0.001 |
| Gender |  |  |  |  |  |  |  |  |  |  |  |
| Female | 1.00 |  | **–** |  | 1.00 |  | **–** |  | 1.00 |  | **–** |
| Male | 2.52 | [2.46–2.58] | <0.001 |  | 0.91 | [0.89–0.94] | <0.001 |  | 1.94 | [1.87–2.01] | <0.001 |
| Age |  |  |  |  |  |  |  |  |  |  |  |
| 40-44 | 1.00 |  | **–** |  | 1.00 |  | **–** |  | 1.00 |  | **–** |
| 45-49 | 2.02 | [1.86–2.20] | <0.001 |  | 2.02 | [1.80–2.28] | <0.001 |  | 1.59 | [1.46–1.75] | <0.001 |
| 50-54 | 3.69 | [3.41–4.00] | <0.001 |  | 4.03 | [3.60–4.51] | <0.001 |  | 2.34 | [2.14–2.55] | <0.001 |
| 55-59 | 6.25 | [5.78–6.75] | <0.001 |  | 7.71 | [6.93–8.58] | <0.001 |  | 3.25 | [2.97–3.54] | <0.001 |
| 60-64 | 10.2 | [9.48–11.0] | <0.001 |  | 13.2 | [11.8–14.6] | <0.001 |  | 4.51 | [4.14–4.91] | <0.001 |
| 65-69 | 15.5 | [14.4–16.7] | <0.001 |  | 21.4 | [19.3–23.7] | <0.001 |  | 5.95 | [5.47–6.47] | <0.001 |
| 70-74 | 22.8 | [21.2–24.5] | <0.001 |  | 27.6 | [24.9–30.6] | <0.001 |  | 7.42 | [6.83–8.07] | <0.001 |
| 75-79 | 30.9 | [28.7–33.3] | <0.001 |  | 33.7 | [30.4–37.4] | <0.001 |  | 8.17 | [7.49–8.90] | <0.001 |
| 80-84 | 37.1 | [34.4–40.1] | <0.001 |  | 36.4 | [32.7–40.5] | <0.001 |  | 8.66 | [7.89–9.52] | <0.001 |
| 85+ | 41.5 | [38.3–44.9] | <0.001 |  | 29.3 | [26.1–32.9] | <0.001 |  | 7.53 | [6.74–8.41] | <0.001 |


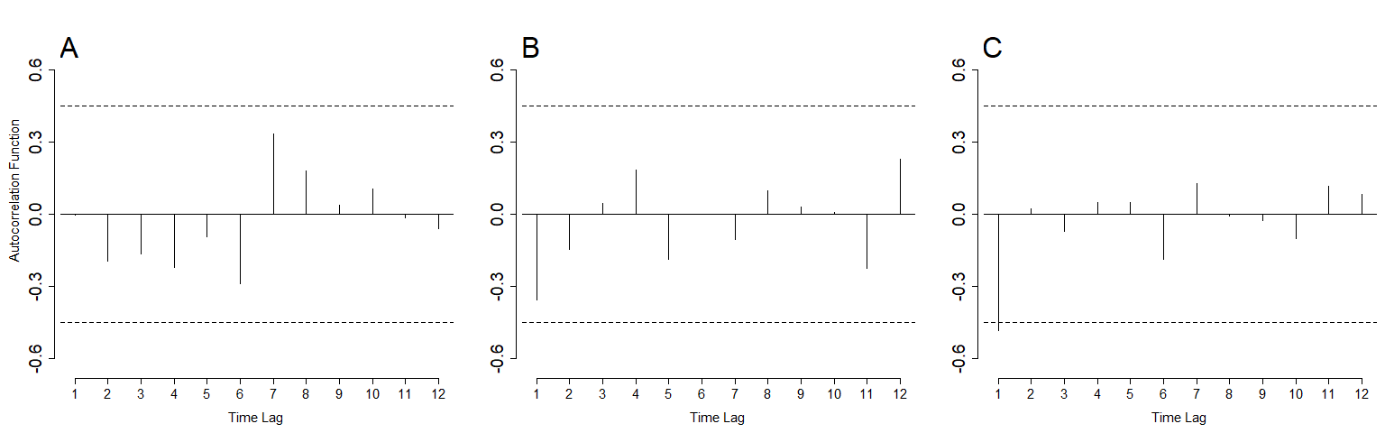


**Figure S1.** Autocorrelations of the residuals. A: bladder cancer, B: carcinoma of the renal pelvis and other urinary organs, C: kidney cancer. Dashed line: 95% confidence interval for the estimated autocorrelation.


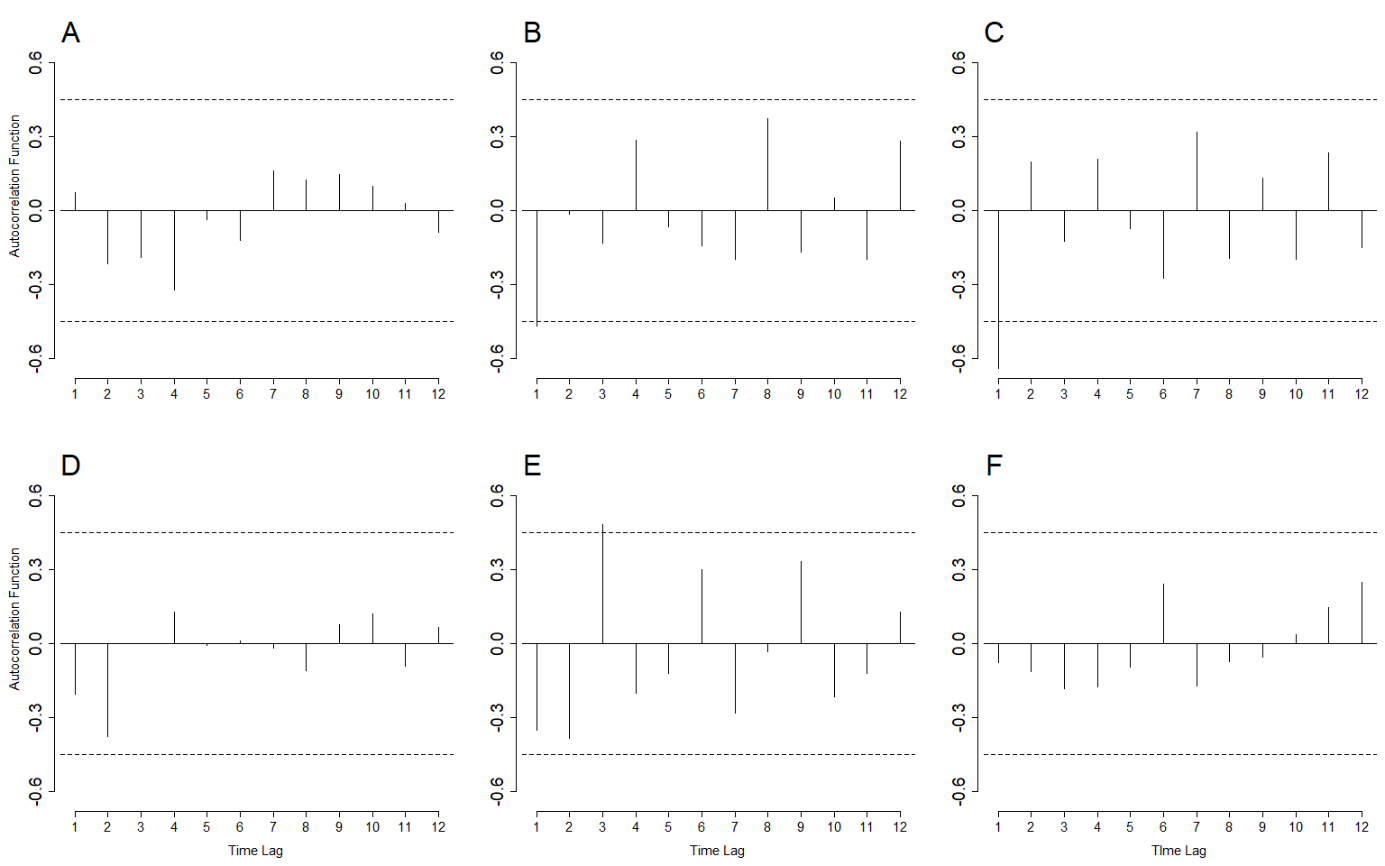


**Figure S2.** Autocorrelations of the residuals by sex. A: bladder cancer in men, B: carcinoma of the renal pelvis and other urinary organs in men, C: kidney cancer in men, D: bladder cancer in women, E: carcinoma of the renal pelvis and other urinary organs in women, F: kidney cancer in women. Dashed line: 95% confidence interval for the estimated autocorrelation.


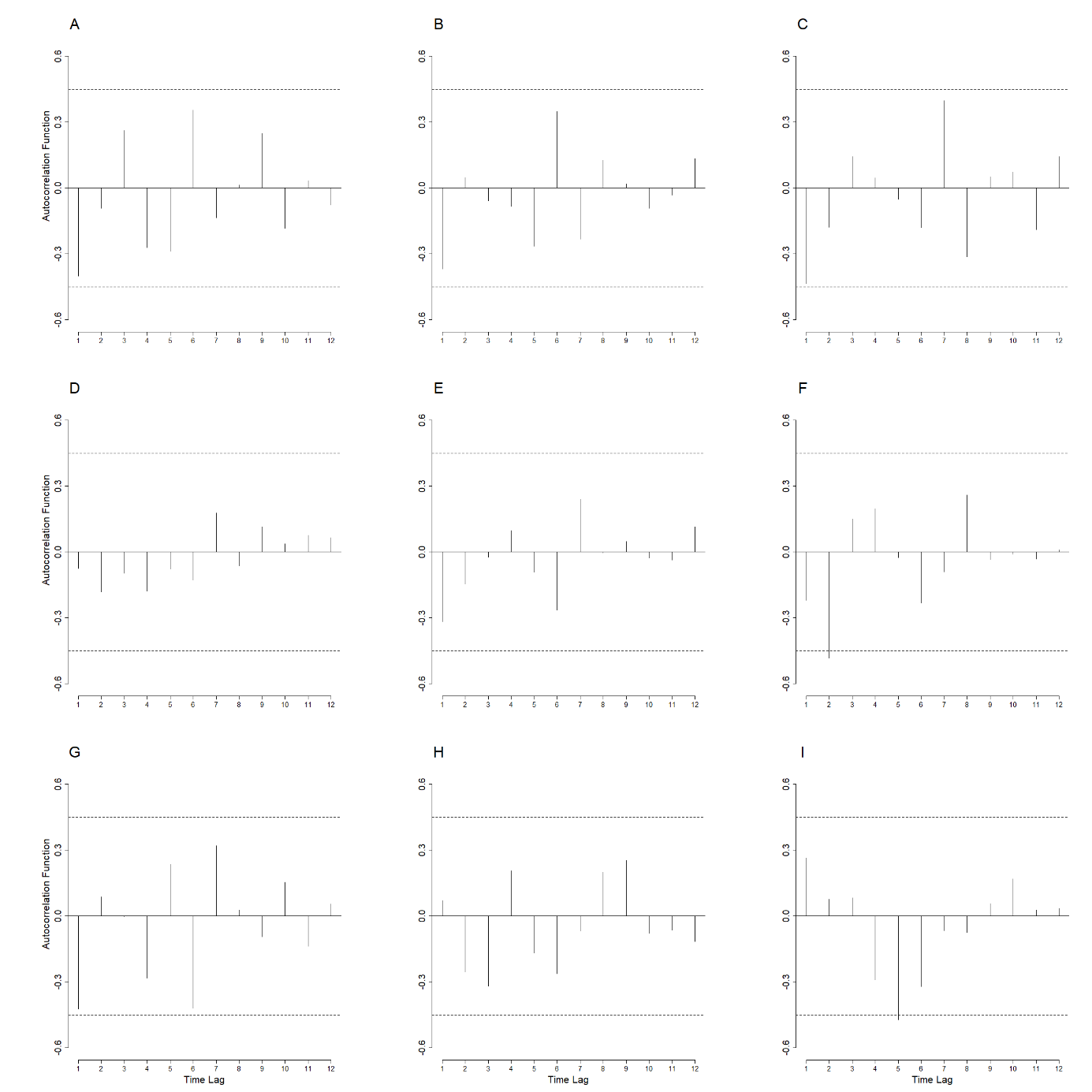


**Figure S3.** Autocorrelations of the residuals by age. A: 40-59 years bladder cancer, B: 40-59 years carcinoma of the renal pelvis and other urinary organs, C: 40-59 years kidney cancer, D: 60-79 years bladder cancer, E: 60-79 years carcinoma of the renal pelvis and other urinary organs, F: 60-79 years kidney cancer, G:
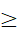
80 bladder cancer, H:
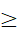
80 carcinoma of the renal pelvis and other urinary organs, I:
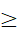
80 kidney cancer. Dashed line: 95% confidence interval for the estimated autocorrelation.


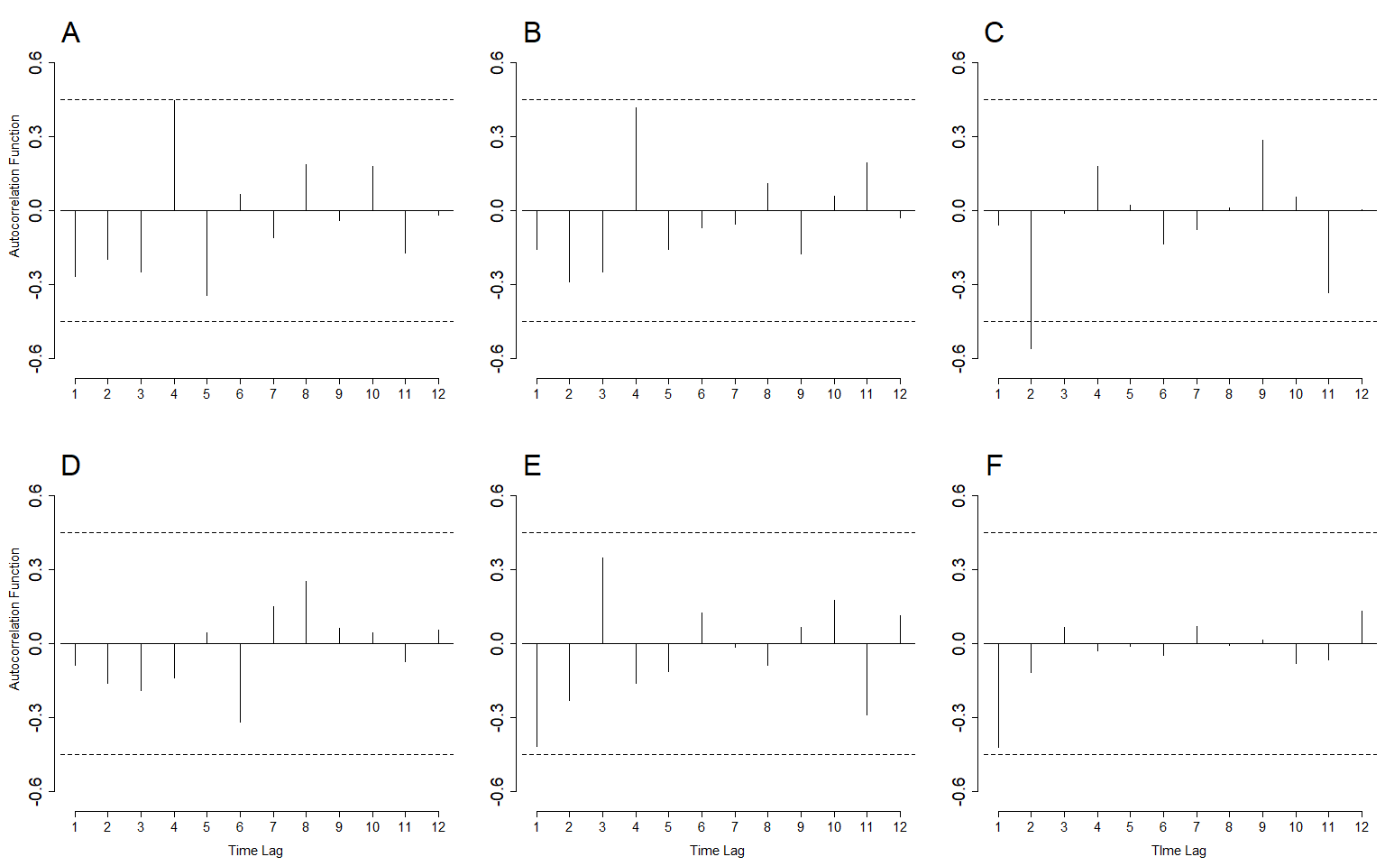


**Figure S4.** Autocorrelations of the residuals by region. A: bladder cancer in blackfoot disease-endemic areas, B: carcinoma of the renal pelvis and other urinary organs in blackfoot disease-endemic areas, C: kidney cancer in blackfoot disease-endemic areas, D: bladder cancer in other areas, E: carcinoma of the renal pelvis and other urinary organs in other areas, F: kidney cancer in other areas. Dashed line: 95% confidence interval for the estimated autocorrelation.


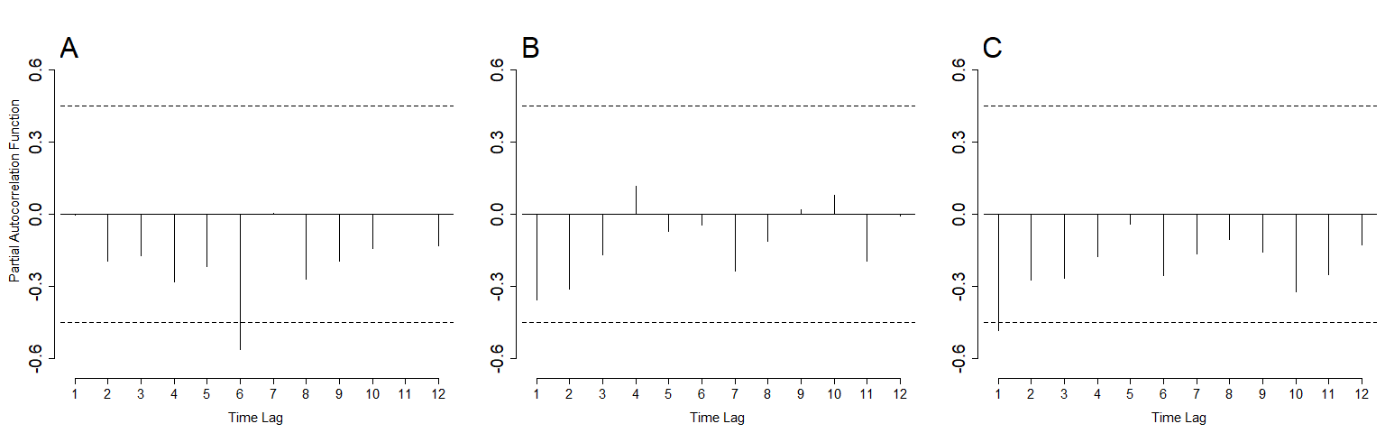


**Figure S5.** Partial autocorrelations of the residuals. A: bladder cancer, B: carcinoma of the renal pelvis and other urinary organs, C: kidney cancer. Dashed line: 95% confidence interval for the estimated partial autocorrelation.


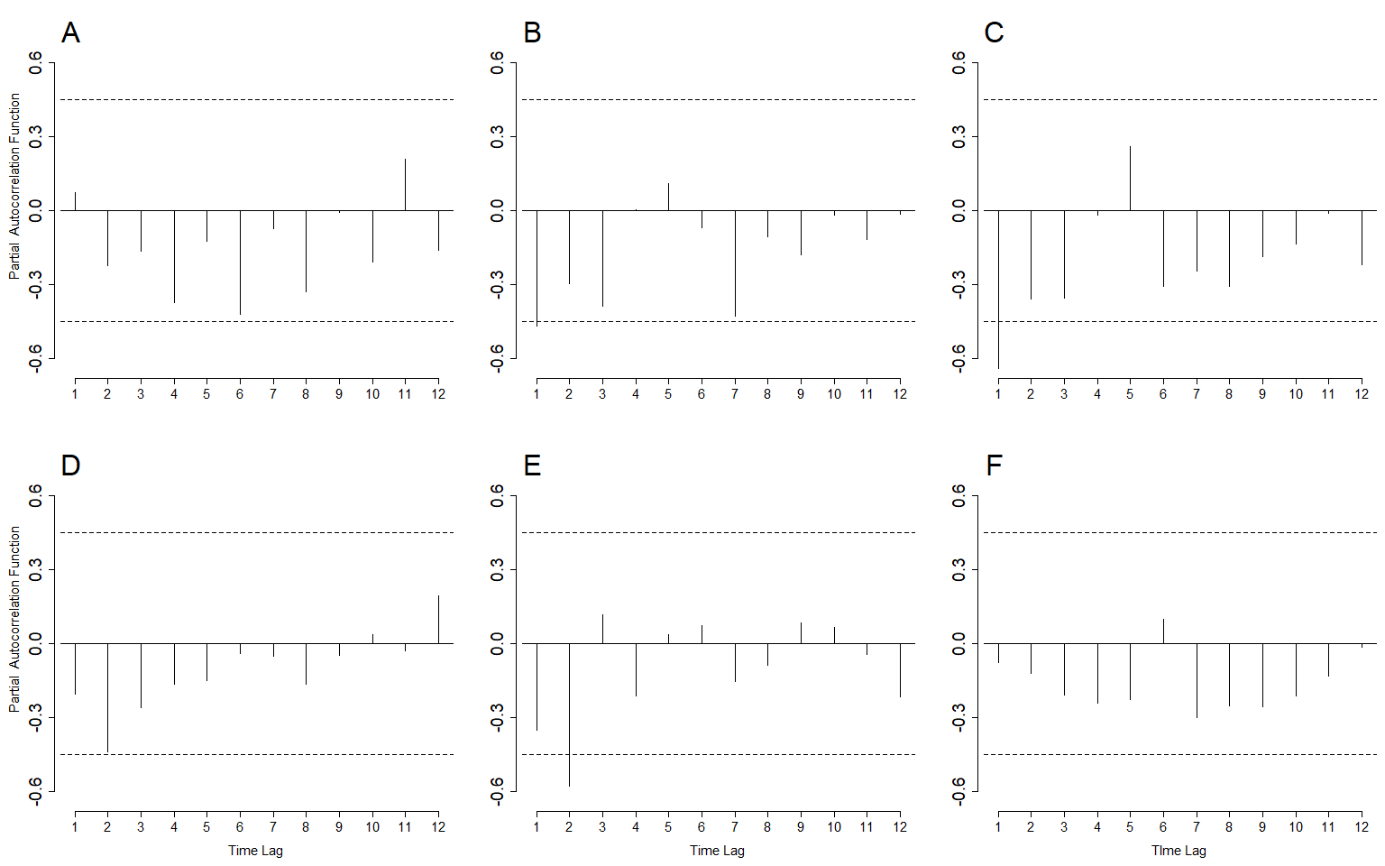


**Figure S6.** Partial autocorrelations of the residuals by sex. A: bladder cancer in men, B: carcinoma of the renal pelvis and other urinary organs in men, C: kidney cancer in men, D: bladder cancer in women, E: carcinoma of the renal pelvis and other urinary organs in women, F: kidney cancer in women. Dashed line: 95% confidence interval for the estimated partial autocorrelation.


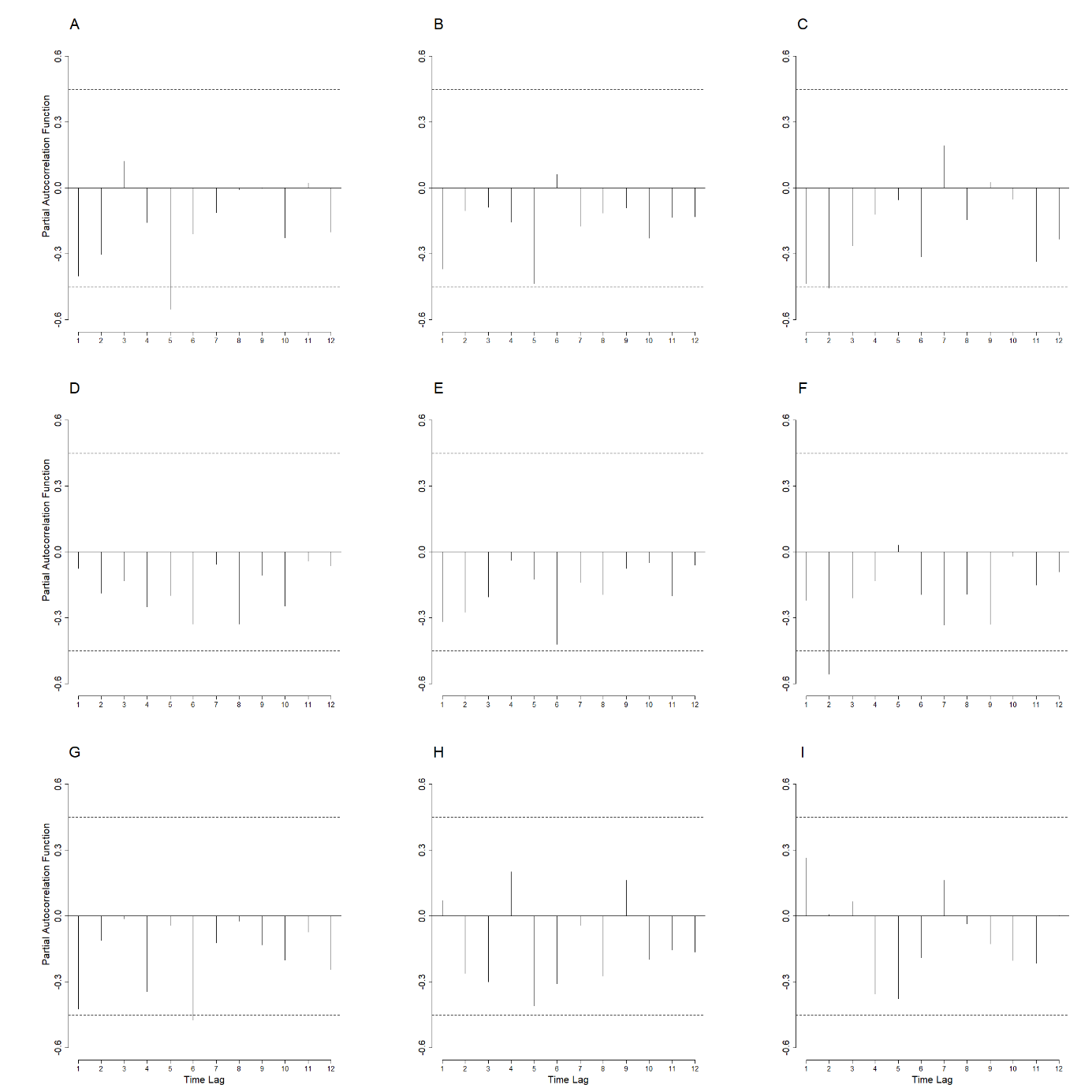


**Figure S7.** Partial autocorrelations of the residuals by age. A: 40-59 years bladder cancer, B: 40-59 years carcinoma of the renal pelvis and other urinary organs, C: 40-59 years kidney cancer, D: 60-79 years bladder cancer, E: 60-79 years carcinoma of the renal pelvis and other urinary organs, F: 60-79 years kidney cancer, G:
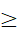
80 bladder cancer, H:
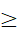
80 carcinoma of the renal pelvis and other urinary organs, I:
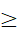
80 kidney cancer. Dashed line: 95% confidence interval for the estimated partial autocorrelation.


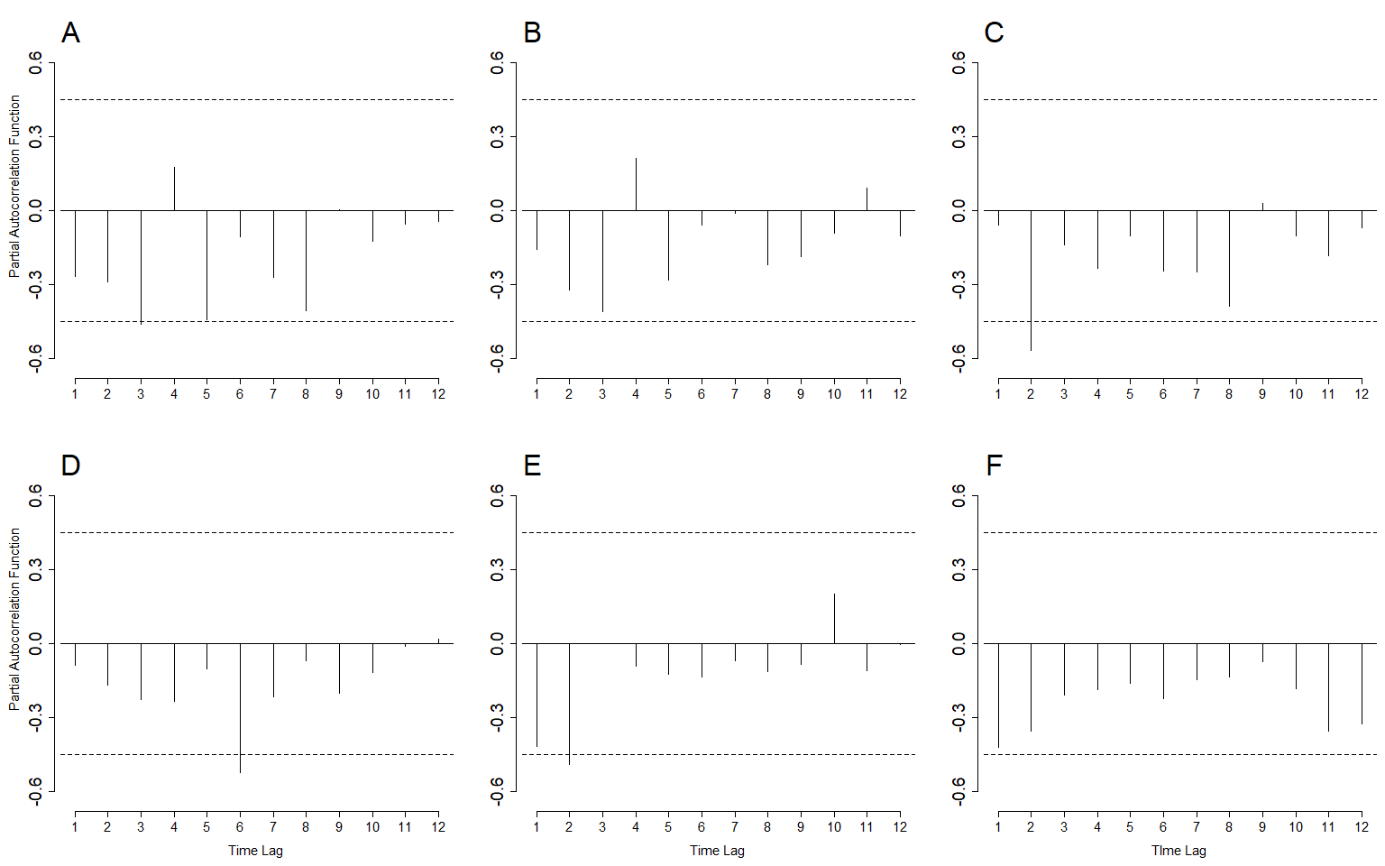


**Figure S8.** Partial autocorrelations of the residuals by region. A: bladder cancer in blackfoot disease-endemic areas, B: carcinoma of the renal pelvis and other urinary organs in blackfoot disease-endemic areas, C: kidney cancer in blackfoot disease-endemic areas, D: bladder cancer in other areas, E: carcinoma of the renal pelvis and other urinary organs in other areas, F: kidney cancer in other areas. Dashed line: 95% confidence interval for the estimated partial autocorrelation.


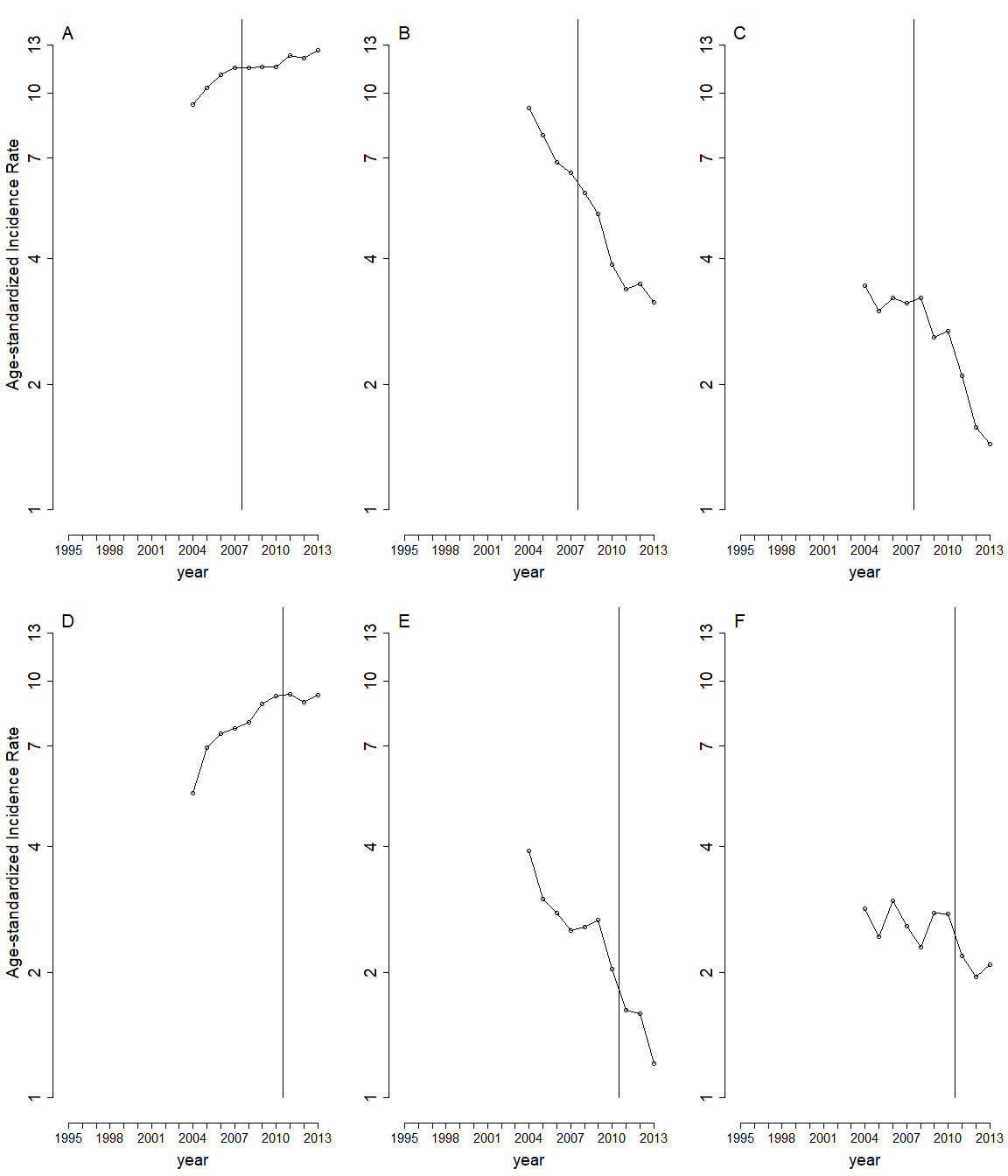


**Figure S9.** Long-term trends in age-standardized incidence rates per 100,000 population by grade. A: high-grade bladder cancer, B: low-grade bladder cancer, C: bladder cancer with unknown grade, D: high-grade carcinoma of the renal pelvis and other urinary organs, E: low-grade carcinoma of the renal pelvis and other urinary organs, F: carcinoma of the renal pelvis and other urinary organs with unknown grade. Circles: age-standardized incidence rates per 100,000 population. Vertical lines: change-of-slope time points.
